# Sexual Behaviour and HPV Infections in 18 to 29 Year Old Women in the Pre-Vaccine Era in the Netherlands

**DOI:** 10.1371/journal.pone.0003743

**Published:** 2008-11-17

**Authors:** Charlotte H. Lenselink, Willem J. G. Melchers, Wim G. V. Quint, Annelies M. J. Hoebers, Jan C. M. Hendriks, Leon F. A. G. Massuger, Ruud L. M. Bekkers

**Affiliations:** 1 Department of Obstetrics and Gynaecology, Radboud University Nijmegen Medical Centre, Nijmegen, the Netherlands; 2 Department of Medical Microbiology, Nijmegen University Centre for Infectious Diseases, Nijmegen, the Netherlands; 3 DDL Diagnostic Laboratory, Voorburg, the Netherlands; 4 Department of Epidemiology and Biostatistics, Radboud University Nijmegen Medical Centre, Nijmegen, the Netherlands; Karolinska Institutet, Sweden

## Abstract

**Background:**

Infection with Human Papillomavirus (HPV) is a necessary event in the multi-step process of cervical carcinogenesis. Little is known about the natural history of HPV infection among unscreened young adults. As prophylactic vaccines are being developed to prevent specifically HPV 16 and 18 infections, shifts in prevalence in the post vaccine era may be expected. This study provides a unique opportunity to gather baseline data before changes by nationwide vaccination occur.

**Methods and Principal Findings:**

This cross-sectional study is part of a large prospective epidemiologic study performed among 2065 unscreened women aged 18 to 29 years. Women returned a self-collected cervico-vaginal specimen and filled out a questionnaire. All HPV DNA-positive samples (by SPF_10_ DEIA) were genotyped using the INNO-LiPA HPV genotyping assay. HPV point prevalence in this sample was 19%. Low and high risk HPV prevalence was 9.1% and 11.8%, respectively. A single HPV-type was detected in 14.9% of all women, while multiple types were found in 4.1%. HPV-types 16 (2.8%) and 18 (1.4%) were found concomitantly in only 3 women (0.1%). There was an increase in HPV prevalence till 22 years. Multivariate analysis showed that number of lifetime sexual partners was the most powerful predictor of HPV positivity, followed by type of relationship, frequency of sexual contact, age, and number of sexual partners over the past 6 months.

**Conclusions and Significance:**

This study shows that factors independently associated with HPV prevalence are mainly related to sexual behaviour. Combination of these results with the relative low prevalence of HPV 16 and/or 18 may be promising for expanding the future target group for catch up vaccination. Furthermore, these results provide a basis for research on possible future shifts in HPV genotype prevalence, and enable a better estimate of the effect of HPV 16-18 vaccination on cervical cancer incidence.

## Introduction

Upcoming mass vaccination with Human Papillomavirus (HPV) vaccines will most certainly change HPV epidemiology. Monitoring these changes on population level may prove crucial in assessing the effect of mass vaccination and overall HPV vaccine efficacy. In the Netherlands, girls aged 12 years will be vaccinated as of September 2009, and the catch-up vaccination (girls aged 13 to 16 years) will probably start in the first part of 2009.

Until now, only a limited number of large studies have investigated HPV epidemiology in female adolescents and young female adults. Even fewer studies have investigated HPV epidemiology in relation to past en present sexual behaviour.

Genital infection with the Human Papillomavirus (HPV) is the most common sexually transmitted infection (STI) among young sexually active women [1]. Most sexually active women (>50%) have been genitally infected by one or more HPV types at some point in their life [2]. Fourteen HPV genotypes are associated with cervical cancer development and are therefore called high-risk (hr-HPV). Of these hr-HPV genotypes, hr-HPV 16 and 18 are related to 70% of all cervical cancers. Therefore, prophylactic vaccines against these two HPV types have been developed. It has been estimated that the best results of prophylactic vaccination will be achieved by vaccinating women before they become genitally infected i.e. sexually active. Presently, vaccination programs are being started in many countries around the world, targeting 9 to 16 year old girls [3], [4]. Additionally, catch-up vaccination of already sexually active women is under consideration in many countries in order to get a faster decrease in cervical cancer incidence.

Estimates of HPV infections among asymptomatic women around the world range from 2% to 44% [5]–[8]. The wide variation in prevalence is largely explained by differences in sensitivity of the HPV-DNA assay used, differences in age, or differences in other characteristics of the populations studied [2], [7].

Additionally, little is known about risk factors for acquiring genital HPV in young female adults. Therefore, further assessment of risk factors like sexual behaviour is important. Knowledge of baseline, i.e. pre-vaccination, epidemiology of type specific HPV infections in relation to sexual behaviour is important in order to decide whether catch-up vaccination may be beneficial. After nationwide implementation of the prophylactic HPV vaccine, HPV epidemiology will most likely change due to expected decreases in HPV 16-18 prevalence and incidence, as well as possible changes in other types occurring due to cross protection of the vaccine. Due to these shifts, prevalence and incidence of other HPV types may increase and therefore may change the oncogenicity of these types.

Therefore, this study, conducted before the nationwide introduction of HPV vaccines, provides a unique opportunity to determine baseline data on HPV prevalence in 18 to 29 year old women in the Netherlands. Additionally, no regular cervical cancer screening is performed in this age group, as the Cervical Screening Program starts at the age of 30 years. This study is part of a large prospective epidemiologic study conducted to study the dynamics of HPV infections, in particular HPV 16/18, and to get more insight in specific risk factors for acquiring genital HPV, like past and present sexual behaviour. These results provide a basis for understanding possible future shifts in genotypes, and presumably enable a better estimate of the effect of HPV 16/18 vaccination on cervical cancer incidence.

## Methods

### Study population and study design

This cross-sectional study is part of a large prospective epidemiologic study performed among 2065 unscreened women aged 18 to 29 years. Women were recruited between June and September 2007, using different advertisements, as well as active recruitment sites, and posters at general practices in the city regions of Arnhem, Nijmegen, and Den Bosch, the Netherlands. Furthermore, advertisement on the internet were used, which were accessible in the whole of the Netherlands. Of the 2297 women who responded to the advertisements, 2065 (89.9%) consented with the study, returned the cervico-vaginal swab specimens, and filled out the questionnaire. Written informed consent was obtained from all participants. This study was approved by the Local Medical Ethics Committee.

### Specimen Collection and Processing

All women were asked to fill out a questionnaire and to self-collect a cervico-vaginal sample in the privacy of their own home. Women received an explanatory letter, an informed consent form, a questionnaire, and a self-sample kit by mail. The self sample kit contained a collection device (a small brush packaged in an individual sterile cover, Vibabrush®, Rovers Medical Devices Oss, the Netherlands), a collection tube containing medium (SurePath^tm^, Tripath Imaging®, Inc., Burlington NC, U.S.A.), instructions how to perform the cervico-vaginal self-sample (written and in cartoon), and a return package consisting of a leak-proof seal bag, absorption sheet, and a reclosable plastic return envelope (easyslider, Transposafe Systems Holland BV). In brief, participants were instructed to wash their hands before opening the brush cover, to hold the brush by the end of the handle, to insert the brush approximately 7 cm into the vagina (similar to inserting a tampon), to gently turn the brush 5 times, and to place the top of the brush in the collection tube. The collection tube was closed, and enclosed in the seal-bag. Finally, the collection tube was placed in the return envelope, together with the questionnaire, and sent to the Department of Obstetrics and Gynaecology for further processing and HPV assessment. The samples were stored at room temperature.

### Questionnaire

In this study we used a questionnaire consisting of two parts. The first part was composed of questions regarding socio-demographic variables like educational level, religion, smoking, medication use, contraceptive use, and ethnicity. Race and ethnicity were self-reported into different categories. The second part consisted of questions regarding sexual behaviour to gain insight in risk factors for acquiring genital HPV. Results of HPV detection were correlated to past and present sexual behaviour. Sex was defined as vaginal, oral, and/or anal sex. For women who had at least 1 lifetime sex partner, additional questions were asked on age at first sexual contact, age of first sex partner, number of sex partners before the age of sixteen, lifetime number of sex partners, number of sex partners in the past 6 months, gender of sex partners, frequency of sexual contact, condom use, and history of sexually transmitted infections (STI).

### HPV DNA Detection and Genotyping

Broad-spectrum HPV DNA amplification was performed using a short PCR fragment assay (SPF_10_-LiPA HPV detection/genotyping assay, SPF_10_ system version 1, manufactured by Labo Biomedical Products bv, Rijswijk, The Netherlands). This assay amplifies a 65-bp fragment of the L1 open reading frame and allows detection of at least 43 different HPV types [9]–[12]. The SPF_10_ PCR was performed with a final reaction volume of 50 μl containing 10 μl of the isolated DNA sample, 10 mmol/liter Tris-HCl (pH 9.0), 50 mmol/liter KCl, 2.0 mmol/liter MgCl_2_, 0.1% Triton X-100, 0.01% gelatin, 200 μmol/liter of each deoxynucleoside triphosphate, 15 pmol each of the forward and reverse primers tagged with biotin at the 5′end, and 1.5 U of AmpliTaq Gold (Perkin-Elmer). The mixture was incubated for 9 minutes at 94°C, 40 cycles of 45 s at 45°C, and 40 cycles of 45 s at 72°C, with a final extension of 5 minutes at 72°C. Each experiment was performed with a separate positive and negative PCR control. The presence of HPV DNA was determined by hybridization of SPF_10_ amplimers to a mixture of general HPV probes recognizing a broad range of HPV genotypes, in a microtiter plate format, as described previously [9]–[12].

All HPV DNA-positive samples (by SPF_10_ DEIA) were genotyped using the INNO-LiPA HPV genotyping assays.

The 28 oligonucleotide probes that recognize 25 different types were tailed with poly(dT) and immobilized as parallel lines to membrane strips (Labo Bio-Medical Products B.V., Rijswijk, The Netherlands). The HPV genotyping assay was performed as described previously [9]. Samples that tested positive using the DNA enzyme immunoassay but that showed no results on the LiPA strip were considered to be HPV X type, i.e. genotypes not available on the LiPA strip. Low risk HPV (lr-HPV) types were defined as HPV type 6,11, 34, 40, 42, 43, 44, 53, 54, 55, 58, 66, 70, 74, and “X”; and hr-HPV types as HPV 16, 18, 31, 33, 35, 39, 45, 51, 52, 56, 59, 68, 73, and 82.

### Statistical Analysis

All women who completed the questionnaire and submitted a swab for HPV evaluation were included in the final analysis (n = 2065).

The Chi-Square test was used to test associations between demographic variables or behavioural characteristics and HPV. Differences of medians of continuous variables between the groups were analysed using non-parametric tests (Mann-Whitney).

In univariate and multivariate analysis, data of some variables were grouped due to small numbers and/or to gain a better overview. We grouped ethnicity into two groups: Dutch and not Dutch. Lifetime number of partners and number of partners in the past six months were divided into four categories, and frequency of sexual contact was grouped into five categories. Years of being sexually active (i.e. sexual age) ranged from 0 to 23 years, the category “0” years consisted of women who became sexually active in the past year. Because of the small numbers, 0 and 1 year were combined as well as 13 to 23 years. Chlamydia, genital warts, Syphilis, Gonorrhea, Genital Herpes, and HIV were defined as STI. In further statistical analysis previous STI's were defined as yes or no.

Variables found to be significantly related to HPV infection by univariate analyses were entered into a multiple logistic regression model with forward selection procedures to identify variables that contributed independently to the probability of HPV prevalence.

Participants with missing data on variables included in the multivariate analysis were excluded. In all tests, p values <0.05 were regarded statistically significant.

Statistical analyses were performed using SAS (8.0) and SPSS 14.1 (Chicago, Illinois).

## Results

### Socio-demographic characteristics

The age distribution and socio-demographic characteristics of the 2065 participants are summarised in table 1. Many women attended higher vocational training or University in past or presence (n = 1545, 75.6%). Of all women, 622 (30.3%) were single and 1431 (69.7%) were involved in a relationship. Only 69 women (3.4%) reported an ethnicity other than Dutch, including “other European” 1.1% (n = 23), Caribbean 0.7% (n = 15), Turkish 0.2% (n = 4), Asian 0.6% (n = 12), African 0.2% (n = 3), and “other” 0.6% (n = 12). Because of these small numbers, they were divided into two groups: Dutch (96.6%, n = 1981), and “other” (3.4%, n = 69).

**Table 1:** HPV prevalence by demographic variables among all women.

	Sample size (n)	HPV Prevalence (n)	P value*
**Overall**	2065	393 (19.0%)	-
**Age (in years)**			<0.001*
18	142	12 (8.5%)	
19	173	19 (11.0%)	
20	190	24 (12.6%)	
21	185	30 (16.2%)	
22	187	41 (21.9%)	
23	185	24 (13.0%)	
24	186	43 (23.1%)	
25	182	44 (24.2%)	
26	186	41 (22.0%)	
27	168	52 (31.0%)	
28	172	37 (21.5%)	
29	109	26 (23.9%)	
**Ethnicity** °	2050		0.73*
Dutch	1981	378 (19.1%)	
Other	69	12 (17.4%)	
**Education** ∧	2044		0.424*
Lower secondary/Lower vocational training	71	13 (18.3%)	
Higher Secondary/Vocational training	428	72 (16.8%)	
Higher vocational training/University	1545	303 (19.6%)	
**Current smoking**	2054		<0.001*
Yes	406	114 (28.1%)	
No	1648	277 (16.8%)	
**Using OCC**	2061		0.758*
Yes	1459	275 (18.8%)	
No	602	117 (19.4%)	
**Living with parents**	2052		0.008*
Yes	357	59 (14.0%)	
No	1695	340 (20.1%)	
**Relationship**	2053		<0.001*
Married	125	7 (5.6%)	
Living together	483	73 (15.1%)	
LAT¤	823	177 (21.5%)	
Single	622	134 (21.5%)	
**Sexual activity ever**			<0.001*
Yes	1947	389 (20%)	
No	116	4 (3.4%)	

HPV+ if one or more genotypes(high risk as well as low risk) are detected simultaneously.

Sample sizes change because of missing values of the questionnaire.

n = number.

- not applicable.

* by Chi-square test.

○ ethnicity was self-reported.

∧ type of education: group of lower secondary education includes 2 women who reported only primary/no education.

¤ Living apart together.

OCC oral contraceptives.

Additionally, the mean age at first sexual contact was 16.7 years, and the mean sexual age (i.e. years of being sexually active) was 6.8 years. Women who were not sexually active yet were significantly more often living with their parents (11.5%, n = 41 versus 4.4%, n = 75, p = <0.001, data not shown).

### Prevalence of HPV infection

**Figure 1:**
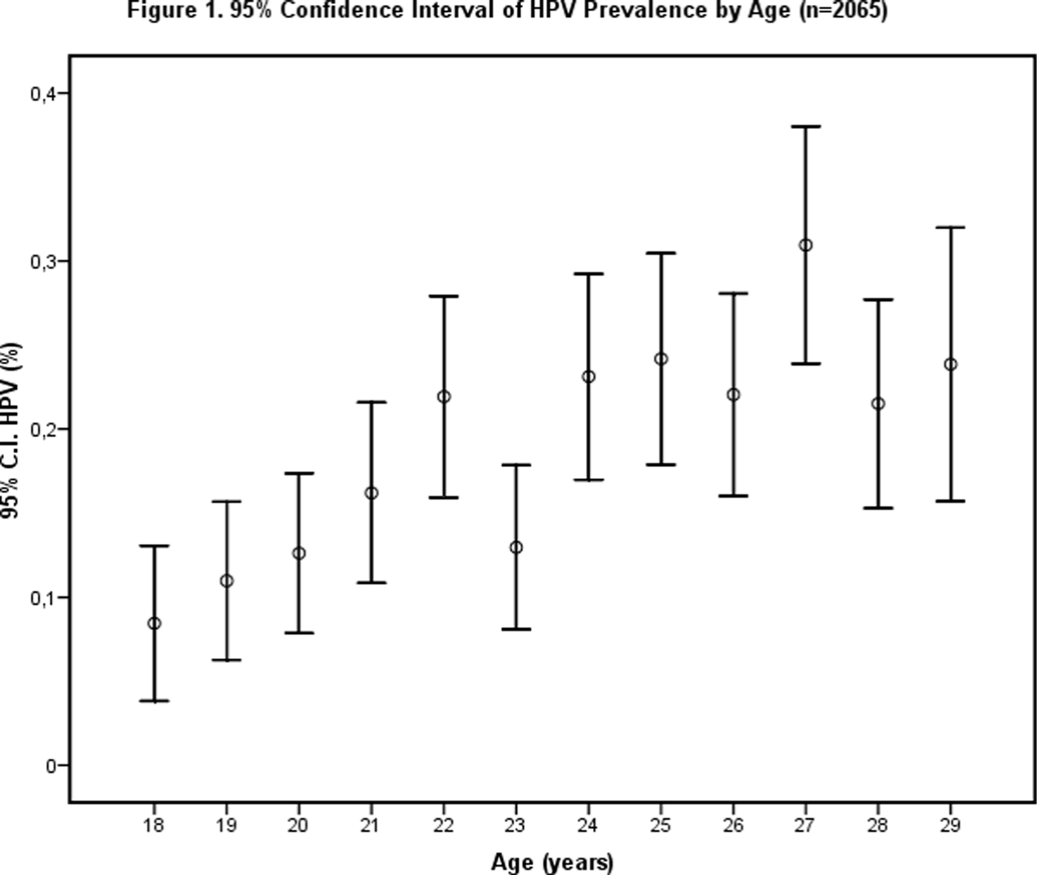
95% Confidence Interval of HPV Prevalence by Age (n = 2065). There was an overall increase in HPV prevalence with age till 22 years, afterwards a plateau phase was reached. A decrease is shown at 23 years and a peak among women aged 27 years, however, as the 95% confidence interval is overlapping with adjacent age groups, the differences were considered accidental findings.

Of the 2065 adequate specimens, 19% (n = 393) tested positive for one or more HPV genotypes. Age-specific prevalence is shown in table 1. There was an overall increase in HPV prevalence with age till 22 years, afterwards a plateau phase was reached. Prevalence of HPV infection showed a decrease at 23 years and a peak among women aged 27 years (13%, n = 24, and 31%, n = 52, respectively). However, as the 95% confidence interval was overlapping with adjacent age groups, the differences were considered accidental findings (see Figure 1).

**Figure 2:**
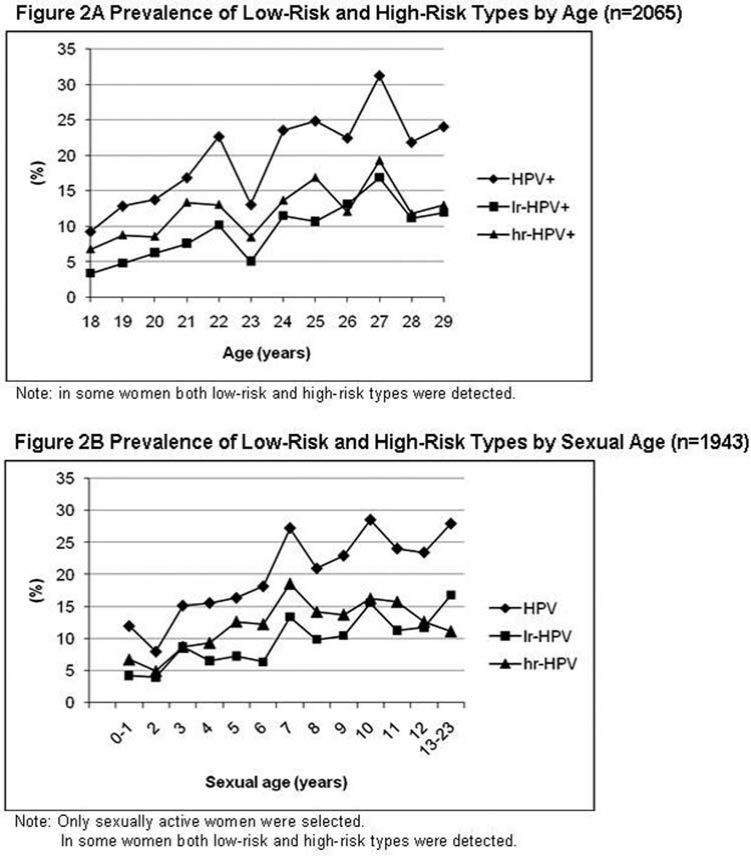
A. Prevalence of Low-Risk and High-Risk Types by Age (n = 2065). Prevalence of overall and both hr- and lr-HPV types showed an almost similar age-distribution. In some women both low-risk and high-risk types were detected. B. Prevalence of Low-Risk and High-Risk Types by Sexual Age (n = 1943). Only sexually active women were selected (n = 1943). Overall HPV prevalence, as well as hr- and lr-HPV prevalence, showed an increase with rising sexual age. However, hr-HPV prevalence decreased from a sexual age of 10 years. In some women both low-risk and high-risk types were detected.

The overall prevalence of hr-HPV types was 11.8% and of lr-HPV types 9.1%, including co-infections. Prevalence of both hr- and lr-HPV types showed an almost similar age-distribution (see Figure 2A).

### Prevalence of specific HPV genotypes

**Figure 3:**
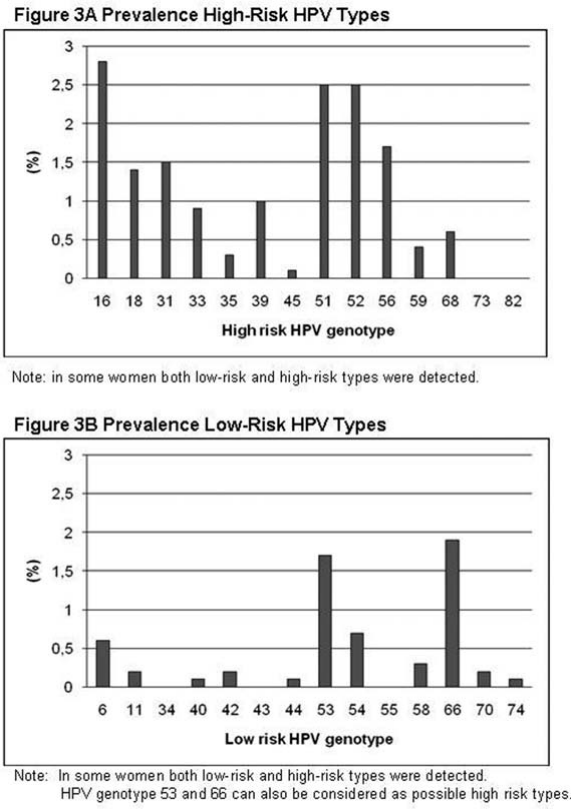
A. Prevalence High-Risk HPV Types. Most common types detected were HPV type 16 (2.8%, n = 57), HPV type 51 (2.5%, n = 51), and HPV type 52 (2.5%, n = 52). In some women both low-risk and high-risk types were detected. B. Prevalence Low-Risk HPV Types. In some women both low-risk and high-risk types were detected. HPV genotype 53 and 66 can also be considered as possible high risk types.

A single HPV-type was detected in 14.9% of all women, while multiple types were found in 4.1% (21.6% of all HPV-positive women). We identified 25 different genotypes, most common types detected were HPV type 16 (2.8%, n = 57), HPV type 51 (2.5%, n = 51), and HPV type 52 (2.5%, n = 52). HPV types 18, 6, and 11, were detected in 1.4% (n = 28), 0.6% (n = 12), and 0.2% (n = 4), respectively (see Figure 3A and 3B). In 3.5% of the women the HPV type could not be specified and was named Lipa X (n = 72). A simultaneous presence of HPV 16 and 18 only occurred in 3 women (0.1%). HPV DNA was detected in 4 women who reported never having had sex. It concerned single infections with HPV type 52, two times HPV type 16, and a co-infection with HPV type 66 and 33.

### Sexually active women

**Figure 4:**
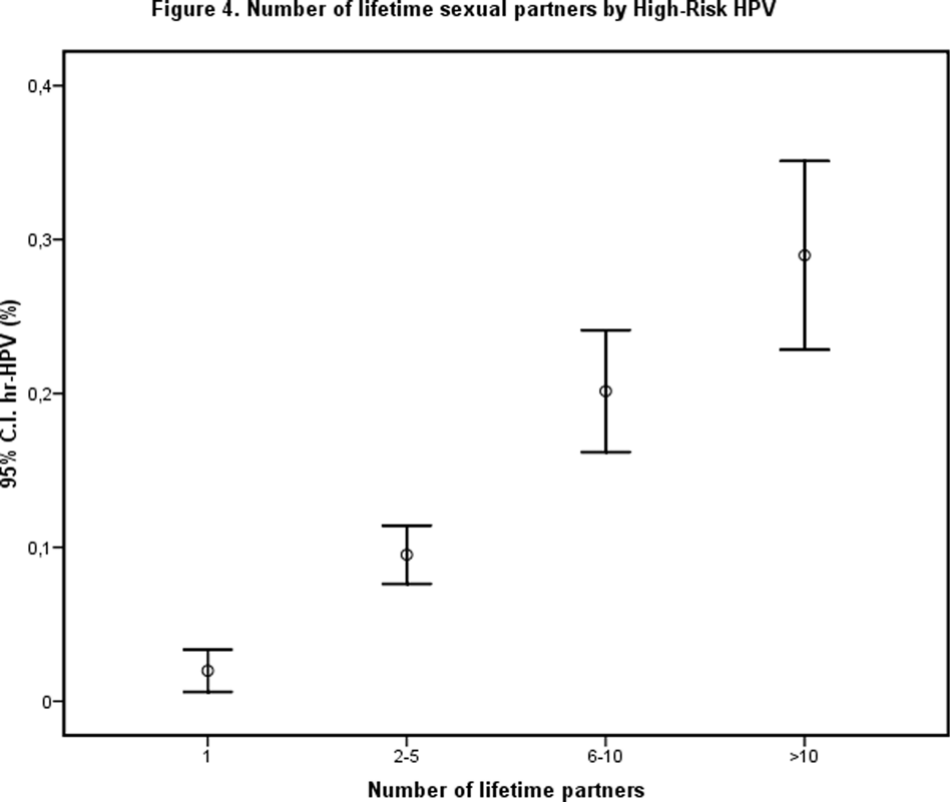
Number of lifetime sexual partners by High-Risk HPV. A higher number of lifetime sexual partners, was significantly associated with overall HPV prevalence as well as hr-HPV prevalence.

When univariate analysis was restricted to sexually active women, factors significantly associated with HPV prevalence were increasing age, current smoking, number of partners in the past 6 months, and years of being sexually active (i.e. sexual age) (see Table 2 and Figure 2B). Sexual age was defined as time interval in years between age at first sexual contact and current age. Furthermore, a higher number of lifetime sexual partners, was significantly associated with overall HPV prevalence as well as hr-HPV prevalence (see Table 2 and Figure 4). Women without an HPV infection tended to be married or living together with their partner. Age at first sexual contact did not show a significant relationship with current HPV prevalence (see Table 2). HPV prevalence, as well as hr- and lr-HPV prevalence, showed an increase with rising sexual age (see Table 2 and Figure 2B). However, hr-HPV prevalence decreased from a sexual age of 10 years (see Figure 2B). Oral contraceptive (OCC) use could not be defined as a risk factor for HPV positivity.

**Table 2:** HPV prevalence and Odds Ratio's for HPV prevalence among sexually active women using univariate analysis and logistic regression.

	n	HPV+	p	OR	(95% C.I.)	p
		n (%)/Median (range)				
**Age (years)**	**1947**	25 (18–29)	<0.001∧	1.097	(1.059;1.136)	<0.001
**Current smoking**	**1936**		<0.001*			
No	1536	274 (17.8%)		0.551	(0.428;0.711)	<0.001
Yes	400	113 (28.3%)		1	(ref)	
**Using OCC**	**1944**		0.272*			
No	528	114 (21.6%)		1.148	(0.898;1.467)	0.272
Yes	1416	274 (19.4%)		1	(ref)	
**Living with parents**	**1934**		0.022*			
No	1619	398 (20.9%)		1.468	(1.055;2.041)	0.023
Yes	315	48 (15.2%)		1	(ref)	
**Relationship**	**1935**		<0.001*			
Married	125	7 (5.6%)		0.216	(0.099;0.471)	<0.001
Living together	483	73 (15.1%)		0.647	(0.480;0.874)	0.004
Single	511	131 (25.6%)		1.254	(0.967;1.625)	0.088
LAT¤	816	176 (21.6%)		1	(ref)	
**Age at first intercourse** ** ** (years)**	**1944**		0.053*			
≤13	45	13 (28.8%)		1.517	(0.719;3.204)	0.274
14–16	935	203 (21.7%)		1.036	(0.688;1.560)	0.866
17–19	803	139 (17.3%)		0.782	(0.514;1.190)	0.251
≥20	161	34 (21.1%)		1	(ref)	
**Lifetime sex partners (number)**	**1938**		<0.001*			
1		17 (4.2%)		0.044	(0.025;0.077)	<0.001
2–5		136 (14.7%)		0.173	(0.125;0.239)	<0.001
6–10		127 (32%)		0.470	(0.334;0.662)	<0.001
>10		107 (50%)		1	(ref)	
**Gender of sex partner(s)**	**1939**		<0.001*			
Male	1829	349 (19.1)		0.393	(0.260;0.594)	<0.001
Female	6	0 (0%)		0.000	(0.000; .)	0.999
Both	104	39 (37.5%)		1	(ref)	
**Sex partners in past 6 months (number)**	**1939**		<0.001*			
0	170	22 (12.9%)		0.140	(0.077;0.254)	<0.001
1	1485	249 (16.8%)		0.190	(0.125;0.288)	<0.001
2	188	66 (35.1%)		0.509	(0.310;0.835)	0.008
>2	99	51 (51.1%)		1	(ref)	
Sexual contact in past 6 months (frequency)	1886		<0.001*			
0	146	19 (13%)		0.461	(0.274;0.775)	0.003
1–6	221	66 (29.9%)		1.312	(0.927;1.857)	0.125
7–24	239	47 (19.7%)		0.754	(0.519;1.096)	0.139
25–54	729	111 (15.2%)		0.553	(0.418;0.732)	<0.001
>54	551	135 (25.4%)		1	(ref)	
Ever diagnosed a STI°?	1940		<0.001*			
No	1755	315 (17.9%)		0.355	(0.257;0.488)	<0.001
Yes	186	71 (38.2%)		1	(ref)	
Condom use	1938		<0.001*			
Never (0%)	924	142 (15.4%)		1.005	(0655;1.541)	0.983
Sometimes (0–50%)	499	134 (26.9%)		2.031	(1.313;3.143)	0.001
Most of times (50–100%)	318	82 (25.8%)		1.923	(1.210;3.055)	0.006
Always (100%)	197	30 (15.2%)		1	(ref)	
**Sexual Age** (years)□	1943	8 (1–13)	<0.001∧	1.096	(1.061;1.133)	<0.001

HPV+ if one or more genotypes are detected simultaneously.

Sample sizes change because of missing values of the questionnaire.

n = number.

95% C.I. Confidence Interval.

p: p-value.

∧ Mann Whitney.

* by Chi-square test.

Ref: reference.

OCC oral contraceptives.

¤ Living apart together.

** below the age of 10 years several cases of sexual abuse were reported.

° STI, Sexually Transmitted Infection.

□ Sexual age in years with 0 and 1 combined as well as sexual age higher than 13.

After logistic regression, age, smoking, number of sexual partners (lifetime and in past 6 months), type of relationship, living with parents, and sexual age were significantly associated with HPV prevalence. Additionally, HPV prevalence was lower among women without a previous STI (Odds Ratio (OR) 0.355, p<0.001, see Table 2), but there was no significant difference between the type of STI's. Women who reported to be non-smokers tested significantly less often positive for HPV than women who reported to be current smokers (17.8% versus 28.3%, OR 0.551, p<0.001, see Table 2). Women not living with their parents tested significantly more often positive for HPV than women who were living with their parents (20.9% versus 15.2%, OR 1.468, 95% C.I. 1.055;2.041, p = 0.02). Age at first sexual intercourse was not significantly related to HPV prevalence, whereas sexual age was (p = 0.053, and p<0.001, respectively).

The analysis was concluded by completing a multivariate regression analysis on all factors that showed a significant relation with HPV in the univariate analysis. The factors independently associated with a risk of being HPV positive were, with an exception for age, mainly related to sexual behaviour (see Table 3). The number of lifetime sexual partners was the most powerful independent predictor of HPV prevalence (p<0.001). Followed by type of relationship (p<0.001), with a significant difference between being married / living together and having a relationship but living apart. This was followed by frequency of sexual contact (p = 0.001), age (p<0.001), and number of sexual partners in past 6 months (p = 0.018), with a protective effect of having a single partner. Sexual age (p = 0.022) was also independently associated with HPV prevalence. Additionally, condom use was not defined as an independent risk factor as it was dependant on age, type of relationship, frequency of sexual contact, and number of sexual partners in the past six months.

**Table 3:** Adjusted Odds Ratio's for HPV prevalence among sexually active women using multivariate logistic regression (n = 1820).

	Adj. OR	(95% C.I.)	p
**Age (years)**	1.160	(1.081;1.246)	<0.001
**Relationship**			<0.001
Married	0.227	(0.098;0.525)	0.001
Living together	0.565	(0.397;0.801)	0.001
Single	1.037	(0.685–1.570)	0.864
LAT¤	1	(ref)	
**Lifetime sex partners** (number)			<0.001
1	0.061	(0.031;0.117)	<0.001
2–5	0.208	(0.139;0.313)	<0.001
6–10	0.512	(0.350;0.748)	<0.001
>10	1	(ref)	
**Sex partners in past 6 months** (number)			0.018
0	0.153	(0.009;2.723)	0.201
1	0.467	(0.278;0.784)	0.004
2	0.674	(0.389;1.169)	0.160
>2	1	(ref)	
**Sexual contact in past 6 months** (frequency)			0.001
0	1.218	(0.073;20.232)	0.886
1–6	0.701	(0.431;1.142)	0.160
7–24	0.541	(0.345;0.848)	0.008
25–54	0.513	(0.375;0.703)	<0.001
>54	1	(ref)	
**Sexual Age** (years)□	0.917	(0.851;0.988)	0.022

n = number.

95% C.I. Confidence Interval.

p: p-value.

¤ Living apart together.

Ref: reference.

° STI, Sexually Transmitted Infection.

□ Sexual age in years with 0 and 1 combined as well as sexual age higher than 13.

## Discussion

This is the first Dutch HPV epidemiological study conducted among unscreened women aged 18 to 29 years. The point prevalence of HPV DNA in this sample was 19%. Lr- and hr-HPV prevalence were 9.1% and 11.8%, respectively. Hr-HPV types 16 (2.8%) and 18 (1.4%) were found concomitantly in only 3 women (0.1%). These results are comparable with recent studies among young women, although different sampling methods were used [8], [13]–[15]. In this large study, self-collected cervico-vaginal samples were used. Material from self-sampling brushes or vaginal lavages has been proven to be highly representative for the cervical HPV status [16]. HPV point prevalence was linked to sexual behaviour by using questionnaires. As the questionnaires were only provided with a study number, they could be considered as fairly anonymous, inducing high credibility. The number of sexual partners, as well as the type of current relationship were significantly associated with HPV positivity. Several international studies confirm sexual behaviour and a high number of sexual partners as the most important risk factors to contract STI's [5], [15], [17]–[22]. In this study not only the number of sexual partners in the past six months but also number of lifetime sexual partners was independently associated with a higher risk for HPV prevalence. An active HPV infection is likely to be dependent on recent sexual activity and may therefore be acquired recently, whereas latent or persistent infection could be influenced by past sexual behaviour. A higher number of lifetime sexual partners increases the risk of getting infected with one or more HPV types in time. Every HPV infection has its type dependent clearance which takes 8 to 14 months on average. Women, who have not been sexually active recently, i.e. in the past six months, may test positive for HPV. Another explanation for the influence of the sexual past is that latent infections are detected. Detecting a latent infection is dependent on the sensitivity of the technique used. In this study the highly sensitive HPV genotyping test SPF_10_-LIPA is used, which could make it difficult to discriminate between active (i.e. chronic productive infections) and latent infections because of its low threshold value. Therefore, results of this study, showing point prevalences of HPV infections, may be a mixture of latent and active or persistent infections.

Furthermore, multivariate analysis provided insight in the independent risk factors for prevalent HPV infection. The independent risk factors were all related to sexual behaviour, with the exception for age. We found that HPV prevalence increased with age. Studies often show HPV prevalence decreasing towards 30 years. This may be explained by the fact that the Dutch Cervical Screening Program starts at the age of 30 years, and therefore these women are unscreened. Furthermore, we did not study women above the age of 29, and we did not combine different age groups, which may provide another perspective by leveling the differences. Other explanations could be the techniques used or differences in the population studied. The use of contraceptive methods like condoms was influenced by type of relationship. Results of several studies on condom use have been inconsistent partly owing to the fact that different populations have been studied [7], [15], [22], [23]. Furthermore, our multivariate analysis showed no significant relation between HPV positivity and smoking. Results of studies regarding the effect of smoking and HPV have also been inconsistent [8], [21], [24].

No correlation of educational level with HPV prevalence was seen. However, the overrepresentation of women attending university college possibly limits generalization of these findings, and may be influenced by the method of recruitment. Nevertheless, this study provides a unique sample with an equal distribution of women over the age groups of 18 to 29 years, using one of the last opportunities to gather baseline i.e. pre vaccine data. These baseline data enable future study on HPV dynamics. HPV epidemiology will most likely change after vaccination due to expected decreases in HPV 16-18 prevalence and incidence, as well as decreases in other types due to cross protection of the vaccine. These decreases in type specific prevalence and incidence may be substituted by increases in other HPV genotypes.

The relative low point prevalence of HPV 16 and 18, and co-infection with both types in only 0.1%, combined with independent predictors of prevalent HPV infection, may be promising for future catch-up vaccination. These results suggest that it may be possible to expand the future target group for catch up vaccination by including women with a higher age or by targeting women with a low-risk profile.

This study shows that sexual behaviour, especially the number of sexual partners as well as type of current relationship, remain the dominant and individual risk factors for HPV positivity i.e. HPV point prevalence. These unique baseline epidemiological data on HPV prevalence in combination with knowledge of sexual behaviour provide a basis for research on possible future shifts in HPV genotype prevalence, and enable a better estimate of the effect of nationwide HPV 16-18 vaccination on cervical cancer incidence.
